# Dengue and COVID-19: Managing Undifferentiated Febrile Illness during a “Twindemic”

**DOI:** 10.3390/tropicalmed7050068

**Published:** 2022-05-07

**Authors:** Liang En Wee, Edwin Philip Conceicao, Jean Xiang-Ying Sim, May Kyawt Aung, Aung Myat Oo, Yang Yong, Shalvi Arora, Indumathi Venkatachalam

**Affiliations:** 1 Department of Infectious Diseases, Singapore General Hospital, Singapore 169608, Singapore; jean.sim.x.y@singhealth.com.sg (J.X.-Y.S.); indumathi.venkatachalam@singhealth.com.sg (I.V.); 2 Department of Infection Prevention and Epidemiology, Singapore General Hospital, Singapore 169608, Singapore; conceicao.edwin.philip@sgh.com.sg (E.P.C.); may.kyawt.aung@sgh.com.sg (M.K.A.); aung.myat.oo@sgh.com.sg (A.M.O.); yang.yong@sgh.com.sg (Y.Y.); shalvi.arora@sgh.com.sg (S.A.)

**Keywords:** dengue, COVID-19, SARS-CoV-2, undifferentiated febrile illness, antigen testing

## Abstract

Background: During the COVID-19 pandemic, distinguishing dengue from COVID-19 in endemic areas can be difficult, as both may present as undifferentiated febrile illness. COVID-19 cases may also present with false-positive dengue serology. Hospitalisation protocols for managing undifferentiated febrile illness are essential in mitigating the risk from both COVID-19 and dengue. Methods: At a tertiary hospital contending with COVID-19 during a dengue epidemic, a triage strategy of routine COVID-19 testing for febrile patients with viral prodromes was used. All febrile patients with viral prodromes and no epidemiologic risk for COVID-19 were first admitted to a designated ward for COVID-19 testing, from January 2020 to December 2021. Results: A total of 6103 cases of COVID-19 and 1251 cases of dengue were managed at our institution, comprising a total of 3.9% (6103/155,452) and 0.8% (1251/155,452) of admissions, respectively. A surge in dengue hospitalisations in mid-2020 corresponded closely with the imposition of a community-wide lockdown. A total of 23 cases of PCR-proven COVID-19 infection with positive dengue serology were identified, of whom only two were true co-infections; both had been appropriately isolated upon admission. Average length-of-stay for dengue cases initially admitted to isolation during the pandemic was 8.35 days (S.D. = 6.53), compared with 6.91 days (S.D. = 8.61) for cases admitted outside isolation (1.44 days, 95%CI = 0.58–2.30, *p* = 0.001). Pre-pandemic, only 1.6% (9/580) of dengue cases were admitted initially to isolation-areas; in contrast, during the pandemic period, 66.6% (833/1251) of dengue cases were initially admitted to isolation-areas while awaiting the results of SARS-CoV-2 testing. Conclusions: During successive COVID-19 pandemic waves in a dengue-endemic country, coinfection with dengue and COVID-19 was uncommon. Routine COVID-19 testing for febrile patients with viral prodromes mitigated the potential infection-prevention risk from COVID-19 cases, albeit with an increased length-of-stay for dengue hospitalizations admitted initially to isolation.

## 1. Introduction

During the COVID-19 pandemic, several dengue-endemic countries in Asia and South America have experienced concurrent outbreaks of dengue and COVID-19 [1,2,3,4]. In the early stages of illness, dengue and COVID-19 can be difficult to distinguish because clinical and laboratory features may potentially overlap, presenting as undifferentiated fever associated with nonspecific signs and symptoms [2]. Co-occurrence and potential co-infection of these two viral diseases introduces a significant burden on healthcare systems, particularly in tropical countries where arboviral diseases are endemic [3]. During overlapping “twin-demics” of dengue and COVID-19, all cases of undifferentiated febrile illness may need to be managed as COVID-19 until proved otherwise via diagnostic testing, with significant implications on healthcare resources. Misdiagnosis or delay in diagnosis of dengue is also conceivable because of the similarities in clinical manifestations of these two diseases [4,5]. Reliance on diagnostic testing to distinguish these two diseases further strains laboratory capacity, especially in resource-limited settings where molecular testing for SARS-CoV-2 and dengue may be unavailable [2]. Rapid serological tests can play a crucial role in dengue diagnostics, especially in low-resource settings where resource-intensive laboratory tests such as polymerase-chain-reaction (PCR) may not be routinely available [6]. Similarly, rapid-antigen-detection (RAD) testing for SARS-CoV-2 has been introduced as a useful component of hospital triage protocols to guide isolation measures and aid targeted admission [7]. However, RAD tests for SARS-CoV-2 may still yield false-negatives and need to be interpreted cautiously, especially in the context of significant contact history or clinical syndromes compatible with COVID-19 [8]. Similarly, there have been reports of false-positive dengue serology with rapid diagnostic tests (RDTs) in cases of COVID-19 [9], resulting in inadvertent exposure of other healthcare workers (HCWs) and patients [10,11]. The triage of patients presenting with undifferentiated febrile illness poses a potential challenge in tropical countries with co-circulating and nonspecific presentations of dengue infection and COVID-19.

In Singapore, a Southeast Asian tropical city-state, successive pandemic waves of COVID-19 were encountered during an ongoing dengue epidemic. In mid-2020, a surge in COVID-19 infections was reported, corresponding to ongoing outbreaks amongst migrant workers living in communal dormitories [12]. This coincided with the imposition of lockdown measures to reduce community transmission of SARS-CoV-2, shifting working patterns into residences, resulting in increased dengue transmission [13]. Dengue is endemic in tropical Singapore [14]. Early on in the COVID-19 pandemic, recognizing that COVID-19 could potentially manifest as undifferentiated viral fever with minimal respiratory symptoms [15], all patients hospitalized for undifferentiated fever were admitted to designated isolation areas where COVID-19 was first ruled out [16]. However, adopting such a broad approach for isolation triage posed its own difficulties in terms of practicality and costs, with 10% of hospital bed capacity set aside for isolation areas [17]. There were sustainability issues, especially due to strain from successive waves of COVID-19 caused by more infectious SARS-CoV-2 variants, including the SARS-CoV-2 delta variant (B.1.617.2) [18]. At our institution, the largest public hospital in Singapore, a triage strategy of routine SARS-CoV-2 testing at admission triage for all febrile patients was utilized, initially with PCR and subsequently supplemented by RAD testing. We evaluated the success of this strategy over a two-year period.

## 2. Materials and Methods

### 2.1. Institutional Setting and Study Period

The Singapore General Hospital is the largest public tertiary hospital in Singapore, with 1785 beds. The first case of COVID-19 in Singapore, in a traveller from Wuhan, was reported from our institution on 23 January 2020 [17]. Over a two-year study period (January 2020 to December 2021), our hospital’s epidemiology team tracked the number of lab-confirmed cases of dengue and COVID-19 managed in our institution. Cases of dengue were diagnosed using a combination of serology, antigen or PCR for additional confirmatory testing; in our institution, dengue diagnostic tests were ordered at the discretion of the primary physician when a clinical syndrome suggestive of dengue was encountered. Cases of COVID-19 were diagnosed using PCR on various molecular platforms. Aggregated descriptive statistics, including length-of-stay (LoS), admission to isolation areas, and in-hospital mortality, were collected for all dengue inpatients during the COVID-19 pandemic and compared against a 2-year pre-pandemic period (January 2018–December 2019). Potential cases of co-infection with both SARS-CoV-2 and dengue were defined as testing positive for SARS-CoV-2 on PCR, as well as having a positive dengue serology result within 48 h of hospitalization; all potential cases were reviewed to exclude false-positive dengue serology.

### 2.2. Workflow for Patients Presenting with Undifferentiated Fever during the COVID-19 Pandemic

From the onset of the COVID-19 pandemic in January 2020, all patients with fever (defined as a single tympanic temperature of ≥37.8 °C) presenting to our institution were triaged in designated “fever areas” of the emergency department (ED), where HCWs used full personal protective equipment (PPE), comprising N95 respirators, gowns, gloves and eye protection, and infrastructural enhancements were introduced, such as partitions between patient cubicles and more frequent cleaning, to mitigate potential exposure to an unsuspected case of COVID-19 [19]. Basic investigations, including bloods and chest radiographs, were performed routinely for all patients presenting with fever in the ED, to aid in risk stratification. Dengue RDTs were also available in the ED. SARS-CoV-2 testing via PCR was available from the onset of the pandemic. While testing was initially ordered at the discretion of the primary physician based on case-definitions issued by the World Health Organisation (WHO) and our local Ministry of Health (MOH), from April 2020 onward, all admissions with fever were routinely screened for SARS-CoV-2 [16], and from June 2021, all admissions were universally screened for SARS-CoV-2 given large community outbreaks attributed to the SARS-CoV-2 delta variant (B.1.617.2 [18]. This degree of enhanced surveillance for SARS-CoV-2 allowed us to determine with certainty the extent of co-infection with both SARS-CoV-2 and dengue amongst all inpatients in the pandemic period, and detect cases of COVID-19 with false-positive dengue serology on RDTs. Given the significant turnaround time required for SARS-CoV-2 testing via PCR, initially patients with undifferentiated fever and no epidemiological risk for COVID-19 were preferentially admitted to designated isolation areas where patients were nursed either in single rooms or cohort rooms with 2–3 patients to a room (modified from usual norm of 5–6 bedded open-plan cohorted cubicles) [17]. HCWs in these wards used full PPE, comprising N95 respirators, gowns, gloves and eye protection when caring for these patients, until the results of SARS-CoV-2 testing returned [17]. From June 2021 onward, in addition to PCR, RAD testing for SARS-CoV-2 was also carried out in the ED for all admissions, with a turnaround time of 15 min [8]. Patients with a positive RAD result were admitted to negative-pressure single rooms in the isolation ward (IW) for confirmatory PCR testing. Patients with negative antigen tests were still risk-stratified for admission to isolation areas based on epidemiological risk and clinical syndromes.

### 2.3. Dengue Diagnostics

Our institution utilized the SD Bioline Dengue Duo (Abbott Diagnostics, Santa Clara, CA) for dengue diagnostic testing in the ED on blood specimens. This is a commercially available rapid immunochromatographic test that comes in a combo of two joint cassettes, one for nonstructural protein 1 (NS1) antigen (Ag) and another for IgM/IgG. Previous studies have indicated a combined sensitivity of 82.4% (95% CI: 76.8–87.1), with a specificity of 87.4% (95% CI: 82.8–91.2) [6]. Dengue NS1 Ag and IgM test using enzyme-linked immunosorbent assay (EIA) is also available inpatient, which has better sensitivity and specificity but a longer turnaround time due to batch testing. Reverse transcription-PCR for dengue virus from blood and urine specimens is also available at our institution as part of an in-house triplex PCR assay (testing for dengue, chikungunya and zikavirus).

### 2.4. COVID-19 Testing

SARS-CoV-2 testing was initially performed on respiratory specimens (nasopharyngeal, oropharyngeal, sputum or bronchoalveolar lavage specimens) using in-house qualitative real-time RT-PCR assays targeting E gene and ORF1b-nsp14 for SARS-CoV-2 [20]. Subsequently, with the availability of commercial assays, PCR testing was performed using the Cepheid Xpert Xpress SARS-CoV-2 assay or the Roche cobas SARS-CoV-2 test [21]. All samples were chemically inactivated for 30 min prior to transfer to the GeneXpert Infinity (Cepheid) in biosafety level 2 containment, or cobas 6800 System (Roche) in biosafety level 2 plus containment, for the SARS-CoV-2 tests. RAD testing for SARS-CoV-2 was performed using the Veritor SARS-CoV-2 antigen rapid test kit (Becton Dickinson, Franklin Lakes, NJ, USA), with a positive percentage agreement of ≥80% and a negative percentage agreement of 99.5% compared to PCR testing [8,22]. Confirmatory SARS-CoV-2 PCR-testing was performed for all positive RAD tests at our institution.

### 2.5. Statistical Methods

Differences in the proportion of dengue hospitalisations requiring high-dependency/intensive-care-unit admission, as well as the proportions of ED admissions presenting with fever during the pre-pandemic and pandemic periods were compared using chi-square test. Length-of-stay for dengue hospitalisations during the pre-pandemic and pandemic periods, and amongst dengue cases initially admitted to isolation areas (versus cases admitted outside of isolation areas) were compared using *t*-test. SPSS (Version 20.0. Armonk, NY, USA: IBM Corp) was used for statistical analysis and a cutoff of *p* < 0.05 was set for statistical significance.

## 3. Results

**Figure 1:**
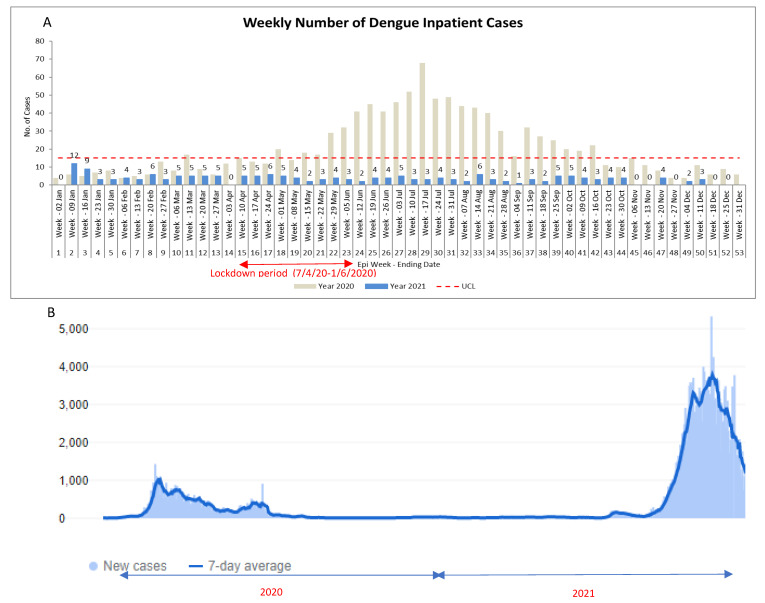
Rates of dengue hospitalisations in a Singaporean tertiary hospital over a 2-year study period during successive waves of community transmission in the COVID-19 pandemic. (**A**) Number of dengue admissions in a Singaporean tertiary hospital from January 2020 to December 2021; (**B**) Epidemic curve of COVID-19 cases in Singapore from January 2020 to December 2021.

Over the COVID-19 pandemic period, a total of 6103 cases of COVID-19 and 1251 cases of dengue were admitted at our institution, comprising a total of 3.9% (6103/155,452) and 0.8% (1251/155,452) of admissions, respectively. A surge in the number of dengue hospitalisations in mid-2020 corresponded closely with the imposition of a community-wide lockdown period in 2020 as part of public health measures for COVID-19 containment. Conversely, despite a surge in COVID-19 cases in end-2021 driven by the SARS-CoV-2 delta variant, there was no surge in dengue hospitalisations in 2021 (Figure 1). Mortality amongst dengue hospitalisations remained low. Pre-pandemic, 0.51% (3/580) of dengue hospitalisations resulted in mortality; during the pandemic period, 0.40% (5/1251) of dengue hospitalisations resulted in mortality. There was no significant difference in mortality amongst dengue hospitalisations during the pandemic period when compared with the pre-pandemic period (incidence-rate-ratio, IRR = 0.77, 95%CI = 0.15–4.98, *p* = 0.716). There was also no significant difference in the odds of high-dependency/intensive-care-unit admission amongst dengue hospitalisations during the pandemic period, when compared with the pre-pandemic period (2.2% (28/1251) vs. 2.9% (17/580), odds-ratio, OR = 0.76, 95%CI = 0.42–1.40). However, average length-of-stay for dengue inpatients during the pandemic period was 7.53 days (standard-deviation, S.D = 7.30), compared with 6.27 days (S.D = 9.59) during the pre-pandemic period; the difference was statistically significant (difference in means = 1.27 days, 95%CI = 0.47–2.07, *p* = 0.002). Average length-of-stay for dengue cases initially admitted to isolation areas during the pandemic period was 8.35 days (S.D = 6.53), compared with 6.91 days (S.D = 8.61) for dengue cases admitted outside of isolation areas; the difference was statistically significant (difference in means = 1.44 days, 95%CI = 0.58–2.30, *p* = 0.001). Pre-pandemic, only 1.6% (9/580) of dengue cases were admitted initially to isolation areas; in contrast, during the pandemic period, 66.6% (833/1251) of dengue cases were initially admitted to isolation areas while awaiting the results of SARS-CoV-2 PCR-testing, due to epidemiological risk (e.g., contact with COVID-19 cases) or overlapping clinical syndromes.

While undifferentiated fever (≥37.8 °C) accounted for a significant proportion of ED admissions, the proportion of ED admissions presenting with fever decreased significantly in the pandemic period, compared to the pre-pandemic period. During the COVID-19 pandemic, 9.0% (8976/99,784) of ED admissions had concomitant fever, compared with 14.3% (15,097/105,435) of ED admissions in the pre-pandemic period (OR = 0.59, 95%CI = 0.57–0.61, *p* < 0.001). In the pre-pandemic period, dengue accounted for only 3.8% (573/15,097) of fever cases admitted via the ED, compared with 11.3% (1018/8976) during the pandemic period (OR = 3.24, 95%CI = 2.92–3.60, *p* < 0.001. In contrast, COVID-19 accounted for 6.1% (6103/99,784) of ED admissions and 17.2% (1548/8976) of fever cases admitted via the ED during the pandemic period. Amongst patients diagnosed with COVID-19, 25.3% (1548/6103) presented with fever (≥37.8 °C) at ED triage. In contrast, fever was present amongst 81.3% (1018/1251) of patients diagnosed with dengue at ED triage during the COVID-19 pandemic. The odds of concomitant fever amongst ED admissions diagnosed with COVID-19 were lower compared to ED admissions diagnosed with dengue (OR = 0.08, 95%CI = 0.07–0.09, *p* < 0.001).

A small proportion (15.9%, 974/6103) of PCR-confirmed COVID-19 cases were concurrently tested for dengue due to a compatible overlapping clinical syndrome. A total of 23 cases of PCR-proven COVID-19 infection with positive dengue serology were identified over the 2-year pandemic period; the large majority of these cases were deemed to have false-positive dengue serology on subsequent review (Table 1). 

Only 2 cases were deemed to have COVID-19 URTI with probable dengue coinfection (NS1-positive; compatible clinical syndrome with fever, myalgia and thrombocytopenia); both cases were managed in isolation from admission due to epidemiological risk factors for COVID-19. Amongst the remaining 21 cases of PCR-proven COVID-19 infection with likely false-positive dengue IgM, only 2 cases were managed outside of isolation areas initially; there was no evidence of onward healthcare-associated transmission to exposed HCWs or patients (Table 1). The remaining cases were isolated from onset due to either epidemiological risk factors or a positive RAD test for SARS-CoV-2, which prompted pre-emptive isolation despite a positive dengue IgM and a potential alternative diagnosis for undifferentiated fever.

## 4. Discussion

During the COVID-19 pandemic, successive waves of both COVID-19 and dengue in a dengue-endemic country placed significant burden on healthcare services; almost 5% of admissions at our institution were concomitantly diagnosed with either COVID-19 or dengue over a 2-year pandemic period. Other studies attributed an increase of over 37.2% in dengue cases from baseline to the introduction of social distancing measures aimed at curbing the spread of SARS-CoV-2 in Singapore; [13] indeed, lockdown measures during the COVID-19 pandemic coincided with a spike in dengue hospitalisations at our institution. This further exacerbated the diagnostic challenge posed by undifferentiated febrile illness during a “twindemic” of both COVID-19 and dengue, as both illnesses could potentially present with febrile syndromes. Over the 2-year pandemic period, COVID-19 and dengue together accounted for almost 30% of patients admitted from our hospital’s ED with fever. Due to infection prevention challenges posed by SARS-CoV-2 and turnaround time required for diagnostic PCR-testing, a large proportion of dengue cases diagnosed via point-of-care testing in our hospital’s ED still required admission to isolation areas while awaiting the return of SARS-CoV-2 PCR-testing. Pre-pandemic, ≤2% of dengue cases required initial admission to isolation areas while awaiting the return of diagnostic testing for other infections; in contrast, during the pandemic period, two-thirds of dengue cases were admitted initially to isolation areas. While there was no significant difference in mortality or odds of requiring high-dependency/intensive-care amongst dengue inpatients at our institution during the pandemic, there was a significant increase in length-of-stay, compared with the pre-pandemic period. This was potentially attributed to the requirement for isolation while awaiting the result of SARS-CoV-2 PCR-testing.

The requirement for isolation of febrile cases with positive dengue serology was driven by concern regarding COVID-19 cases masquerading as dengue with false-positive IgM as well as shared clinical and laboratory features between COVID-19 infection and dengue [1]. This is a clinical conundrum unique to dengue-endemic countries grappling with the COVID-19 pandemic; indeed, the first reports of patients incorrectly diagnosed with dengue due to a false-positive dengue rapid serological test who were subsequently diagnosed with COVID-19 originated from Singapore [9]. Misdiagnosis of COVID-19 as dengue with failure to isolate such patients could potentially trigger outbreaks in healthcare settings. Cases of potential nosocomial transmission have been reported amongst HCWs attending to such patients without appropriate PPE, due to the misplaced reassurance of a false-positive dengue serology test [10,11]. In addition, dengue and SARS-CoV-2 co-infection has been reported, providing an additional diagnostic challenge [23]. However, there is little information on the prevalence of dengue and SARS-CoV-2 co-infection; our experience suggests that both false-positive dengue IgM and co-infection with dengue are uncommon scenarios for COVID-19 infection, even in a dengue-endemic county. Over a two-year period, despite widespread availability of diagnostic testing for both dengue and COVID-19, only 21 cases of COVID-19 infection with false-positive dengue IgM and 2 cases of dengue and SARS-CoV-2 co-infection were identified at our centre, forming <0.5% of all COVID-19 cases admitted over the same time period. The infection prevention consequences of COVID-19 cases masquerading as dengue with false-positive IgM need to be balanced against the low likelihood, in practice, of encountering such cases, as well as the resources required to pre-emptively isolate all patients with undifferentiated febrile illness while awaiting the return of PCR-testing for COVID-19. Point-of-care tests, such as RAD testing for SARS-CoV-2, may potentially offer the clinician some additional reassurance with a faster clinical turnaround, though issues of sensitivity and specificity remain [22].

The limitations of our study are as follows. As this was a single-centre study, direct extrapolation of our observations to other contexts is difficult; nevertheless, the long study period allowed us to observe the prevalence of both dengue and COVID-19 at our institution through successive pandemic waves, given the seasonal nature of both dengue and COVID-19 infection. Prolonged length-of-stay during the pandemic period might have been due to other contributory factors associated with the challenges of care delivery during a pandemic, not just isolation requirements; nevertheless, throughout the pandemic our hospital continued to function as normal and did not require temporary closures due to nosocomial COVID-19 outbreaks, in part due to stringent inpatient and HCW surveillance [18]. Despite the stress placed on clinical laboratories during the COVID-19 pandemic [2], diagnostic testing for both COVID-19 and dengue continued to be made available at our institution throughout the pandemic period, with no delay in turnaround times. While false-positive dengue serology could be ruled out via PCR testing, the possibility of cross-reactivity with a different flavivirus could not be completely excluded. However, there were no outbreaks of zikavirus reported in Singapore during the study period, and Japanese encephalitis is not endemic in Singapore. Additionally, the prevalence of dengue may be underestimated since the sensitivity of NS1 detection with rapid diagnostic tests is lower during secondary infections, and dengue PCR was only performed in selected samples to confirm infection.

## 5. Conclusions

During successive COVID-19 pandemic waves in a dengue-endemic country, dengue was established as an alternative diagnosis in a minority of COVID-19 suspects. Coinfection with dengue and COVID-19 was uncommon. A triage strategy of routine COVID-19 testing for febrile patients with viral prodromes was successful in containing the potential infection-prevention risk from COVID-19 cases masquerading as dengue with false-positive IgM. While there was no significant difference in mortality amongst dengue hospitalisations during the pandemic, there was a significant increase in length-of-stay, especially amongst dengue cases initially admitted to isolation while awaiting results of SARS-CoV-2 testing.

## Figures and Tables

**Table 1:** Cases of PCR-proven COVID-19 infection with positive dengue serology at a Singaporean tertiary hospital during the COVID-19 pandemic, 2020–2021.

Case Number	Biodata	Presenting Symptoms	Pulmonary Infiltrates on Chest Radiograph	Thrombocytopenia at Presentation (10^9^/L)	Dengue Tests (Serology and/or PCR)	Diagnosis	Outcome	Infection Prevention Consequences
1	31 yo male	Fever, sore throat, headache, myalgia, ageusia	No	Yes (nadir 109)	NS1 +ve, IgM −ve	COVID-19 URTI with probable dengue coinfection (NS1 +ve)	Full recovery	None. Managed in isolation from admission due to epidemiological risk factors for COVID-19
2	31 yo male	Fever, headache, myalgia, cough	No	Yes (nadir 122)	NS1 –ve, IgM +ve	COVID-19 URTI with likely false-positive dengue IgM	Full recovery	None. Managed in isolation from admission due to epidemiological risk factors for COVID-19
3	38 yo male	Fever, sore throat, headache, myalgia	No	No	NS1 –ve, IgM +ve	COVID-19 URTI with likely false-positive dengue IgM	Full recovery	None. Managed in isolation from admission due to epidemiological risk factors for COVID-19
4	34 yo male	Vomiting, diarrhea	No	No	NS1 –ve, IgM +ve	COVID-19 URTI with likely false-positive dengue IgM	Full recovery	None. Managed in isolation from admission due to epidemiological risk factors for COVID-19
5	29 yo male	Fever, headache, myalgia, cough, diarrhea	No	No	NS1 –ve, IgM +ve	COVID-19 URTI with likely false-positive dengue IgM	Full recovery	None. Managed in isolation from admission due to epidemiological risk factors for COVID-19
6	69 yo female	Fever	Yes	Yes (nadir 120)	NS1 –ve, IgM +ve	COVID-19 URTI with likely false-positive dengue IgM	Full recovery but needed ICU admission	None. Managed in isolation from admission due to epidemiological risk factors for COVID-19
7	38 yo male	Fever, headache, sore throat, myalgia, vomiting, diarrhea	No	No	NS1 –ve, IgM +ve; blood PCR at day 4 of illness –ve	COVID-19 URTI with false-positive dengue IgM (PCR negative)	Full recovery	None. Managed in isolation from admission due to epidemiological risk factors for COVID-19
8	34 yo male	Fever, headache, vomiting, dysgeusia	No	Yes (nadir 125)	NS1 –ve, IgM +ve; blood PCR at day 4 of illness –ve	COVID-19 URTI with false-positive dengue IgM (PCR negative)	Full recovery	**Initially spent 14 hrs outside of isolation.** 11 HCW and 2 inpatient close-contacts, none tested positive for SARS-CoV-2 on 14d surveillance
9	48 yo male	Fever, myalgia	No	Yes (nadir 82)	NS1 –ve, IgM +ve; blood PCR at day 7 of illness –ve	COVID-19 URTI with false-positive dengue IgM (PCR negative)	Full recovery	**Initially spent 14.5 hrs outside of isolation.** 10 HCW and 1 inpatient close-contacts, none tested positive for SARS-CoV-2 on 14d surveillance
10	43 yo male	Asymptomatic	No	Yes (nadir 100)	NS1 –ve, IgM +ve	COVID-19 URTI with likely false-positive dengue IgM	Full recovery	None. Managed in isolation from admission due to epidemiological risk factors for COVID-19
11	26 yo male	Cough, rhinorrhea	No	Yes (nadir 110)	NS1 –ve, IgM +ve	COVID-19 URTI with likely false-positive dengue IgM	Full recovery	None. Managed in isolation from admission due to epidemiological risk factors for COVID-19
12	30 yo male	Fever, myalgia	No	Yes (nadir 75)	NS1 +ve, IgM –ve	COVID-19 URTI with probable dengue coinfection (NS1 +ve)	Full recovery	None. Managed in isolation from admission due to epidemiological risk factors for COVID-19
13	49 yo male	Fever, cough, sore throat, rhinorrhea	Yes	No	NS1 –ve, IgM +ve	COVID-19 pneumonia with likely false-positive dengue IgM	Full recovery	None. Managed in isolation from admission due to epidemiological risk factors for COVID-19
14	89 yo male	Myalgia	No	No	NS1 –ve, IgM +ve	COVID-19 URTI with likely false-positive dengue IgM	Full recovery	None. Managed in isolation from admission due to positive rapid-antigen-detection test for COVID-19
15	73 yo male	Myalgia	No	Yes (nadir 100)	NS1 –ve, IgM +ve	COVID-19 URTI with likely false-positive dengue IgM	Full recovery	None. Managed in isolation from admission due to positive rapid-antigen-detection test for COVID-19
16	57 yo male	Fever, cough, dyspnea	Yes	Yes (nadir 105)	NS1 –ve, IgM +ve	COVID-19 pneumonia with likely false-positive dengue IgM	Full recovery	None. Managed in isolation from admission due to positive rapid-antigen-detection test for COVID-19
17	68 yo male	Fever, cough	Yes	Yes (nadir 96)	NS1 –ve, IgM +ve	COVID-19 pneumonia with likely false-positive dengue IgM	Full recovery	None. Managed in isolation from admission due to positive rapid-antigen-detection test for COVID-19
18	67 yo male	Fever, cough	No	Yes (nadir 110)	NS1 –ve, IgM +ve	COVID-19 pneumonia with likely false-positive dengue IgM	Full recovery	None. Managed in isolation from admission due to positive rapid-antigen-detection test for COVID-19
19	76 yo male	Fever, dyspnea	Yes	Yes (nadir 105)	NS1 –ve, IgM +ve	COVID-19 pneumonia with likely false-positive dengue IgM	Demised at D32 of illness, required ICU admission	None. Managed in isolation from admission due to epidemiological risk factors for COVID-19
20	57 yo male	Fever, cough, rhinorrhea, sore throat	Yes	No	NS1 –ve, IgM +ve	COVID-19 pneumonia with likely false-positive dengue IgM	Full recovery	None. Managed in isolation from admission as though rapid-antigen-detection test for COVID-19 was negative, patient had epidemiological risk factors for COVID-19
21	65 yo female	Fever, cough	No	Yes (nadir 105)	NS1 –ve, IgM +ve; blood PCR at day 4 of illness –ve	COVID-19 URTI with false-positive dengue IgM (PCR negative)	Full recovery	None. Managed in isolation from admission due to positive rapid-antigen-detection test for COVID-19
22	69 yo male	Fever, cough, dyspnea, diarrhea	Yes	Yes (nadir 106)	NS1 –ve, IgM +ve	COVID-19 pneumonia with likely false-positive dengue IgM	Full recovery	None. Managed in isolation from admission due to epidemiological risk factors for COVID-19
23	56 yo male	Fever, rhinorrhea, maculopapular rash	No	Yes (nadir 52)	NS1 –ve, IgM +ve; blood PCR at day 4 of illness –ve	COVID-19 URTI with false-positive dengue IgM (PCR negative)	Full recovery. Case of acute HIV seroconversion	None. Managed in isolation from admission due to epidemiological risk factors for COVID-19

## Data Availability

The datasets for this study are available from the authors on reasonable request.
